# Size Controlled Copper (I) Oxide Nanoparticles Influence Sensitivity of Glucose Biosensor

**DOI:** 10.3390/s17091944

**Published:** 2017-08-24

**Authors:** Tian Lan, Ahmad Fallatah, Elliot Suiter, Sonal Padalkar

**Affiliations:** 1 Department of Mechanical Engineering, Iowa State University, Ames, IA 50011, USA; tlan@iastate.edu (T.L.); fallatah@iastate.edu (A.F.); esuiter@iastate.edu (E.S.); 2 Microelectronics Research Center, Iowa State University, Ames, IA 50011, USA

**Keywords:** electrodeposition, nanoparticle size, biosensor, glucose

## Abstract

Copper (I) oxide (Cu_2_O) is an appealing semiconducting oxide with potential applications in various fields ranging from photovoltaics to biosensing. The precise control of size and shape of Cu_2_O nanostructures has been an area of intense research. Here, the electrodeposition of Cu_2_O nanoparticles is presented with precise size variations by utilizing ethylenediamine (EDA) as a size controlling agent. The size of the Cu_2_O nanoparticles was successfully varied between 54.09 nm to 966.97 nm by changing the concentration of EDA in the electrolytic bath during electrodeposition. The large surface area of the Cu_2_O nanoparticles present an attractive platform for immobilizing glucose oxidase for glucose biosensing. The fabricated enzymatic biosensor exhibited a rapid response time of <2 s. The limit of detection was 0.1 μM and the sensitivity of the glucose biosensor was 1.54 mA/cm^2^. mM. The Cu_2_O nanoparticles were characterized by UV-Visible spectroscopy, scanning electron microscopy and X-ray diffraction.

## 1. Introduction

Copper (I) oxide (Cu_2_O) is a highly attractive oxide semiconductor due to its unique properties. It is a p-type semiconductor having a direct bandgap of 2 eV. Cu_2_O is a non-toxic material and its starting material, copper, is abundantly available. Furthermore, the fabrication and processing of Cu_2_O is inexpensive. Due to these advantages, Cu_2_O has potential applications in several fields including photovoltaics, catalysis, batteries, gas sensing and biosensing [1,2,3,4,5,6,7,8,9]. In photovoltaics, Cu_2_O presents a promising alternative to silicon and other potential semiconductors. The author, Rai, in a review article has provided a comprehensive overview of Cu_2_O as an appealing material for solar cells including the inexpensive fabrication methods, the construction of a solar cell followed by its performance. This review also highlights the advantages of the Cu_2_O material and some of its drawbacks [4]. It has been demonstrated that Cu_2_O is a potential material for gas sensing. Deng and co-workers have used graphene oxide conjugated with Cu_2_O nanowires for nitrogen dioxide sensing. Here they demonstrated the crystallization of Cu_2_O in the presence of graphene oxide to form nanowires, which were highly anisotropic. These structures show a high performance, as compared to the separate systems of Cu_2_O and graphene oxide [5]. In another application, Cu_2_O was utilized as a photocathode for solar water splitting [10]. Paracchino and co-workers demonstrated a highly efficient Cu_2_O photocathode with the highest recorded photocurrent of −7.6 mA/cm^2^ [10]. The Cu_2_O nanostructures have also been utilized as platforms for biosensing. Zhu and co-workers synthesized Cu_2_O hollow microspheres with the help of polyvinylpyrrolidone [11]. The Cu_2_O hollow microspheres were investigated for biosensing applications and served as an excellent immobilization platform for the DNA probe and enhanced the sensitivity of the DNA biosensor. In a similar study, an enzymatic biosensor was fabricated using graphene oxide, zinc oxide and Cu_2_O [12]. The composite biosensing electrode exhibited enhanced immobilization of glucose oxidase (GOx) enzyme with a linear range of 0.01–2 mM and the detection limit of 1.99 μM.

In all the above studies the Cu_2_O nanostructures were presented in varying morphologies ranging from thin film to nanocubes. Thus, it is pertinent to note that Cu_2_O can be fabricated in several different morphologies. The variations in morphologies has been studied and well documented in the literature. It has been demonstrated that variation in morphology can affect the properties of Cu_2_O including their optical and electronic properties. Radi and co-workers fabricated size and shape controlled Cu-Cu_2_O core shell nanoparticles via electrodeposition on H terminated silicon [13]. The size was varied between 5–400 nm and different shapes including cubic, cuboctahedral, and octahedral were obtained by controlling the deposition time and the electrolyte concentration respectively. Zhang and co-workers synthesized nearly monodispersed Cu_2_O nanoparticles by a hydrothermal method [14]. Here, they observed that by carefully changing the reactant concentration the Cu_2_O nanoparticle size, monodispersity and crystallinity can be controlled. In another example, Xu and co-workers synthesized octahedral Cu_2_O nanoparticles with varying edge length from 130 nm to 600 nm [15]. This variation in edge length was carried out by adjusting the molar ratio of the reactants. The absorption properties of these Cu_2_O nanoparticles also varied and thus demonstrated improved ability in photodegradation of methyl orange compared to cubic Cu_2_O nanoparticles. Feng and co-workers demonstrated the formation of hollow spherical and octahedral Cu_2_O nanocrystals in the presence of EDA and sodium hydroxide [16]. The change in morphology affected the photocatalytic activity of the Cu_2_O nanocrystals. Thus, size and shape control of the Cu_2_O nanoparticles can alter their properties significantly.

In the present work, a strong control over the size of the Cu_2_O nanoparticle during electrodeposition was demonstrated by utilizing ethylenediamine (EDA) in the electrolytic bath. To the best of our knowledge, this is the first report of size-controlled synthesis of Cu_2_O nanoparticles, using EDA during the electrodeposition technique. The electrodeposition method is facile, inexpensive and scalable [17,18,19]. It can be used for precise control of size and morphology of the depositing species. The Cu_2_O nanoparticles have been synthesized in varying sizes from 54.09 nm to 966 nm. The Cu_2_O nanoparticle electrodes, fabrication by the above mentioned route, were utilized for the first time as platform for glucose biosensing. The current response of the Cu_2_O electrodes indicates that the nanoparticle size has a strong influence on the sensitivity of the glucose biosensor. The Cu_2_O electrodes were characterized by UV-visible spectroscopy (UV-Vis), scanning electron microscopy (SEM) and X-ray diffraction (XRD).

## 2. Materials and Methods

### 2.1. Materials

The chemicals used for the electrodeposition of Cu_2_O were cupric sulfate pentahydrate (CuSO_4_·5H_2_O, ≥98%), ethylenediamine (EDA), lactic acid (C_3_H_6_O_3_, ≥88.5%) and potassium hydroxide (KOH, ≥85.8%). These chemicals were purchased from Fisher Scientific (Hanover Park, IL, USA). The chemicals did not require any further purification and thus were used as purchased. The aqueous solutions were prepared by dissolving the precursor in deionized water. The electrodeposition was performed on a fluorine doped tin oxide (FTO) coated on glass substrate. The size and conductivity of the substrate was 25 mm × 25 mm × 1.1 mm and 6–8 ohm/sq. respectively. The substrate was purchased from University Wafer Inc. (Boston, MA, USA). The chemicals used for cleaning the FTO substrate were acetone (100%, 200 proof), hydrochloric acid (HCl) and nitric acid (HNO_3_).

### 2.2. Fabrication of Cu_2_O Electrode via Electrodeposition

**Figure 1:**
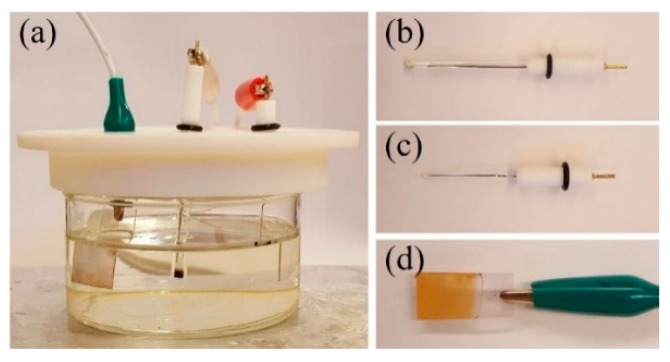
(**a**) Electrodeposition setup, with the reference (**b**), counter (**c**) and working (**d**) electrodes.

The electrodeposition was performed in an electrochemical cell (Figure 1a). For the electrodeposition of Cu_2_O, an Ag/AgCl wire was used as the reference electrode (Figure 1b). A platinum wire of 2 mm diameter served as the counter electrode (Figure 1c) and an FTO substrate was the working electrode (Figure 1d). Prior to electrodeposition, the FTO substrate was sonicated for 10 min in a bath of acetone. It was then cleaned by hydrochloric acid (HCl) followed by nitric acid (HNO_3_) for 2 min each. The substrate was rinsed with deionized water between every cleaning step.

For the electrodeposition of Cu_2_O nanoparticles, the CuSO_4_·5H_2_O precursor was dissolved in deionized water. The aqueous solution was stabilized by the addition of C_3_H_6_O_3_ to the solution. The addition of EDA was carried out after the copper precursor was completely dissolved in solution. The pH of the final solution was adjusted, to 13, by utilizing KOH. The electrodeposition temperature was kept at 50 °C. The duration for electrodeposition was 30 min. During electrodeposition, the applied potential was −0.6 V.

### 2.3. Fabrication of Enzymatic Biosensor

The glucose oxidase (GOx), enzyme was immobilized on the Cu_2_O electrode by electrostatic interaction. Since the isoelectric point (IEP) of GOx is 4.5 and that of Cu_2_O nanoparticles is ~11, the electrostatic interaction is strong leading to successful immobilization [20]. The GOx enzyme solution was prepared by dissolving 1 mg of GOx in 1 mL of 10 mM phosphate buffer saline (PBS) at a pH of 7.4. The immobilization was carried out by drop casting 100 μL of the GOx enzyme solution onto the Cu_2_O electrode and left to dry for 2 h at room temperature. The dried electrode was rinsed with PBS to discard any enzyme that was not immobilized. The Cu_2_O electrode with immobilized GOx was stored in PBS at 4 °C overnight in a refrigerator.

### 2.4. Characterization

The absorption properties of the fabricated Cu_2_O nanoparticles was studied by a UV-Visible spectrometer, which was a Lambda 25 instrument. The morphology of the Cu_2_O nanoparticles was evaluated by scanning electron microscopy (SEM), a FEI Quanta-250 SEM instrument operating at 10 kV accelerating voltage. The composition of the Cu_2_O nanoparticles was investigated by X-ray diffraction (XRD). The XRD instrument was a Siemens D500. The X-ray diffractometer utilized a Cu Kα radiation (λ = 1.5406 Å) at 45 kV and 30 mA, with a scanning range of 20° to 80° and a scan step of 0.05°. The electrodeposition was carried out by using a CHI601E potentiostat from CH Instruments.

## 3. Results and Discussion

### 3.1. Characterization of Electrodeposited Cu_2_O Nanoparticles

**Figure 2:**
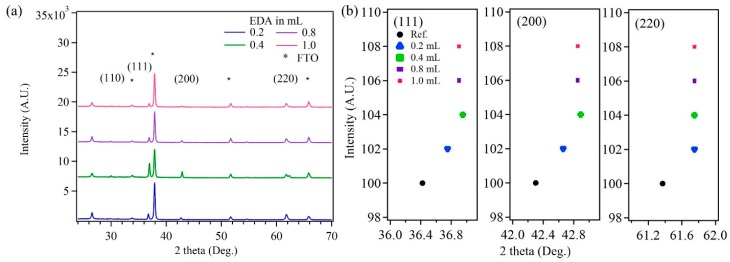
(**a**) XRD plots of Cu_2_O samples with varying ethylenediamine (EDA) contents; (**b**) The right shift in the 2-theta values for (111), (200) and (220) planes.

The composition and crystallinity of the deposited Cu_2_O nanoparticles was characterized by X-ray diffraction. Figure 2a shows XRD plots of Cu_2_O nanoparticles with varying EDA content. The XRD plots were indexed and matched well with the Cu_2_O reference (JCPDS: 05–0667), having a cubic crystal structure. The XRD plots clearly show (111), (200) and (220) peaks for Cu_2_O. The intensity of the XRD peaks decreased with increase in the EDA content in the Cu_2_O samples. It was also observed that the sample thickness reduced with increase in the EDA content. Additionally, it was observed that the peak positions were shifted to higher Bragg values with increase in the EDA content (Figure 2b). This shift can be attributed to the decrease in the lattice parameter with increase in the EDA content. The peak position, in Figure 2b, for the samples with varying EDA content have been offset along the *Y* axis for clear viewing.

**Figure 3:**
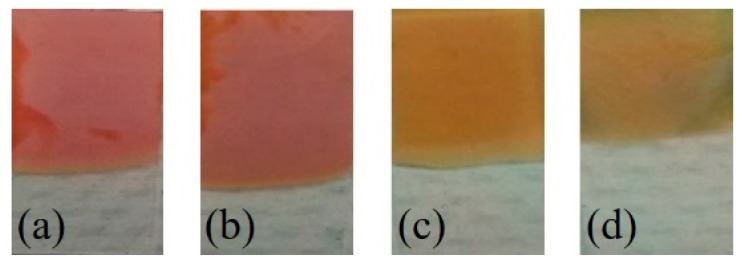
Photographs Cu_2_O samples prepared by electrodeposition in the presence of varying volumes of EDA in electrolytic bath from (**a**) 0.2 mL; (**b**) 0.4 mL; (**c**) 0.8 mL to (**d**) 1 mL.

The photographs of the Cu_2_O nanoparticles electrodeposited on the FTO substrates are shown in Figure 3. These Cu_2_O nanoparticles were prepared with increasing content of EDA solution, from 0.2 mL to 1 mL, in the electrolytic bath during deposition. From these photographs it was clear that there was a distinct difference in the color of the Cu_2_O samples with increasing EDA content during deposition. The Cu_2_O sample with 0.2 mL of EDA appeared red-orange in color (Figure 3a), while the sample with 1 mL of EDA appeared yellow in color (Figure 3d). Thus these photographs suggested that there was a change in the absorption properties of the samples with increase in the EDA content in the deposition process.

**Figure 4:**
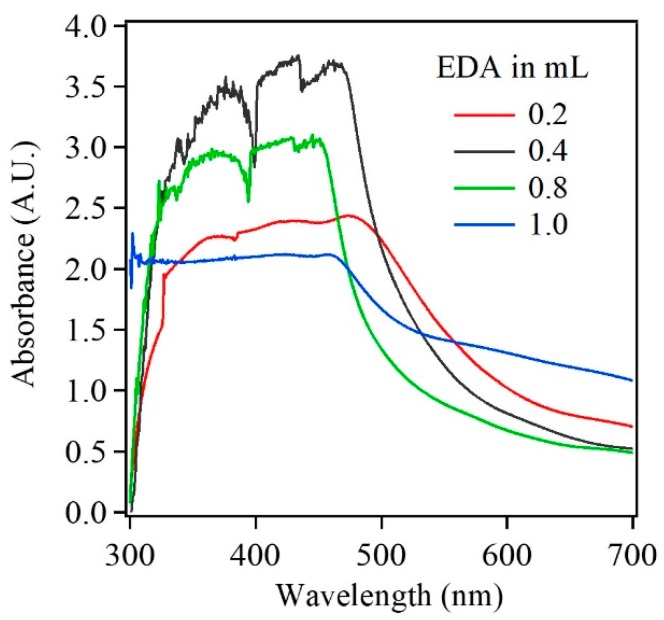
UV-Vis spectra of Cu_2_O nanoparticle with increase in the EDA content from 0.2 mL (red curve), 0.4 mL (black curve), 0.8 mL (green curve) to 1 mL (blue curve).

Additionally, UV-Vis absorption spectra were collected from the Cu_2_O samples to evaluate their absorption properties. Figure 4 shows UV-Vis spectra for Cu_2_O samples with EDA content of 0.2 mL and 1 mL. The absorption between 350 nm and 550 nm was assigned to the inter-band transition in Cu_2_O nanoparticles. Further, the broad band feature around 700 nm was attributed to the localized surface plasmon resonance, which is observed in Cu_2_O nanoparticles [21]. Additionally, a blue shift in the absorption spectra indicated a decrease in the nanoparticle size with increase in the EDA content of the Cu_2_O samples. The differences in the absorption spectra for 0.2 mL and 1 mL EDA can be related to the photographs shown in Figure 2. For Cu_2_O sample with 0.2 mL EDA, the combination of absorption peaks at 510 nm and 700 nm can be related to the red-orange color (Figure 3a,b). As the EDA content was increased to 1 mL, the absorption peak blue shifted to 475 nm and a higher intensity broader peak was observed beyond 600 nm. The absorption peak combination of 475 nm and higher intensity at 700 nm can be related to the yellowish color of the Cu_2_O sample with 1 mL EDA (Figure 3c,d).

**Figure 5:**
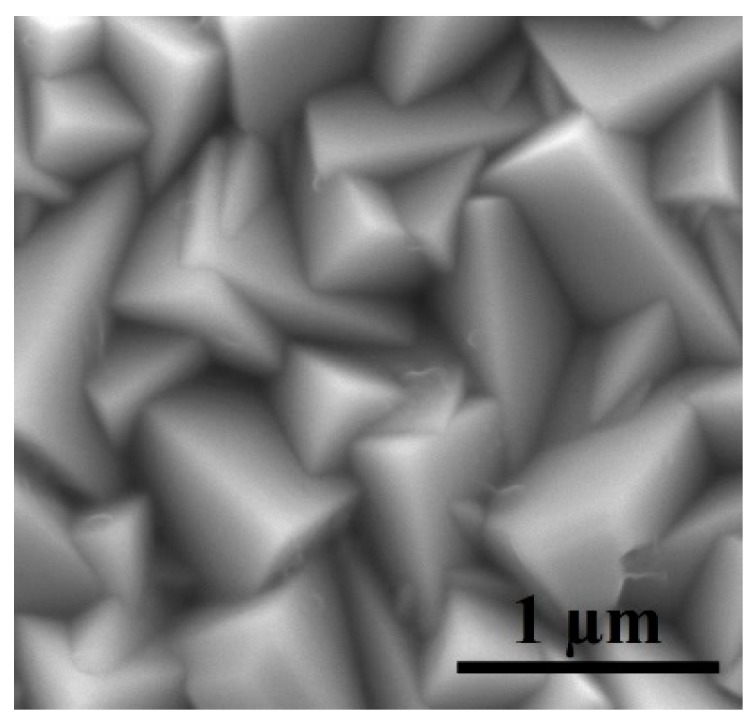
SEM image of Cu_2_O reference sample, fabricated in the absence of EDA.

To further probe the nanoparticles size of the Cu_2_O samples, a series of SEM images were obtained and particle size distribution was calculated. Figure 5 shows the SEM image of Cu_2_O nanoparticles fabricated in the absence of EDA. Here, we observe a cubic structure of the Cu_2_O nanoparticles with size approximately 750 nm.

**Figure 6:**
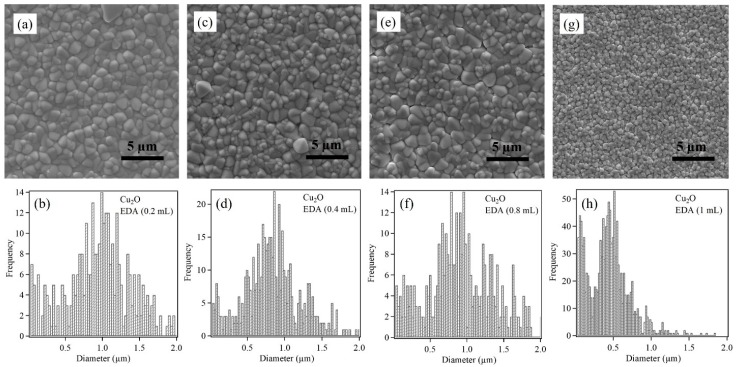
SEM images and size distribution of Cu_2_O nanoparticles with increasing EDA content from (**a**,**b**) 0.2 mL, (**c**,**d**) 0.4 mL, (**e**,**f**) 0.8 mL to (**g**,**h**) 1 mL.

Figure 6 shows SEM images of Cu_2_O oxide samples with increasing EDA content along with their corresponding size distribution. Figure 6a shows Cu_2_O nanoparticles deposited in the presence of 0.2 mL of EDA in the electrolytic bath. The shape of the Cu_2_O nanoparticles appear to be mix of triangular and rhombic shapes. When the EDA content was increased to 0.4 mL the average nanoparticle size decreased. The SEM image in Figure 6c shows a combination of large and small nanoparticles. It was also observed that all the nanoparticles had similar shapes as seen in Figure 6a. Further increase in the EDA content to 0.8 mL did not show any apparent change in the Cu_2_O nanoparticle size and shape (Figure 6e). However, with additional increase in the EDA content to 1 mL, drastic decrease in the nanoparticle size and shape was observed (Figure 6g). Here, a bimodal distribution was observed. This distribution is confirmed by the SEM image for 1 mL EDA sample, which shows small Cu_2_O nanoparticles underneath larger nanoparticles. Moreover, the nanoparticle size distribution appeared to be narrow for both nanoparticle sizes. The nanoparticles were quasi spherical in shape. Table 1 provides the average nanoparticle size for Cu_2_O samples under investigation in the present work.

### 3.2. Cu_2_O Nanoparticles as Biosensing Platform

**Figure 7:**
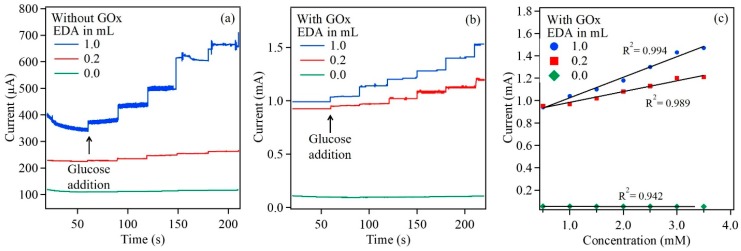
Amperometric response of Cu_2_O/ITO (**a**) and GOx/Cu_2_O/ITO (**b**) electrodes to glucose in 10 mM PBS at applied potential of 0.8 V. Calibration curve for GOx/Cu_2_O/ITO electrode (**c**).

Here, Cu_2_O nanoparticles were utilized for glucose sensing to test whether the fabricated electrodes can serve as a robust and viable biosensing platform. The steady-state amperometric response of the enzymatic biosensor was investigated by the successive addition of equal amounts of glucose in 10 mM PBS, at an applied potential of 0.8 V under constant stirring condition. Amperometric response was first obtained from Cu_2_O reference sample followed by Cu_2_O samples with 0.2 mL and 1 mL EDA. These samples were not treated with GOx. All samples exhibited amperometric response, to the addition of glucose, in the absence of GOx. The amperometric response was higher in samples with EDA. The response and sensitivity increased with EDA content (Figure 7a). In the presence of GOx, the Cu_2_O samples with EDA exhibited an increase in the overall current along with distinct amperometric response. Figure 7b exhibited a rapid and sensitive response to the addition of glucose for the two biosensors fabricated in the presence of EDA. The current response increased with the increase in the glucose concentration at every step. The biosensors also demonstrated a fast current response of <2 s. Additionally, the Cu_2_O nanoparticles with higher concentration of EDA (1 mL) exhibited a total current enhancement compared to sample with 0.2 mL EDA. The reference sample, immobilized with GOx, exhibited the lowest current response compared to the other samples fabricated with EDA. Thus the total current response indicated that the biosensor was more sensitive to increased surface area. Furthermore, the concentration of EDA used during the deposition process eventually influences the sensitivity of the biosensor. Figure 7c shows the calibration curves for the Cu_2_O reference sample and Cu_2_O samples with EDA content of 0.2 mL and 1.0 mL. It is evident that the current increases with glucose concentration, almost linearly from a range of 0.1 mM to 3.5 mM. The sensitivity of the biosensors ranges between 1243.2–1538 μA/cm^2^. mM for Cu_2_O nanoparticles with EDA content of 0.2 mL to 1.0 mL respectively. The affinity of GOx to the substrate, glucose was obtained by calculating the apparent Michaelis–Menten constant with the help of the Lineweaver–Burk equation [22]:

$$\frac{1}{i}=\;\left(\frac{K_{M}^{app}}{i_{max}}\right)\;\left(\frac{1}{C}\right)+\;\frac{1}{i_{max}}$$

where *C* is the glucose concentration, $i_{max}$ and i are the currents for substrate saturation and steady state respectively, during the glucose sensing measurements. From the calculation, $K_{M}^{app}$ was obtained to be 1.00 mM and 1.25 mM for Cu_2_O samples with EDA content of 0.2 mL and 1.0 mL respectively, which indicates good affinity of the immobilized GOx to glucose. The biosensor characteristics obtained in this work were compared to the values in literature, shown in Table 2 [12]. The present work exhibited higher detection limit among other studies in literature. Additionally, a large linear range was obtained. Stability test on the biosensor was also performed. The Cu_2_O with 1 mL EDA sample was tested after 7 days of initial amperometric response. The amperometric response of the EDA sample diminished slightly after 7 days of storage.

From the above characterizations and biosensor investigation of the Cu_2_O nanoparticles it was clear that the nanoparticle size decreased and the density increased with increase in EDA content. Evidently, the optical properties varied with changing EDA content. Additionally, the XRD data provided evidence of decreasing lattice parameter with increasing EDA content. Furthermore, the sensitivity was enhanced with decrease in nanoparticle size. Thus, it was evident that EDA played an important role in controlling the size, density, optical and biosensing properties of the Cu_2_O nanoparticles. It was therefore pertinent to understand the influence of EDA on the final outcome of the Cu_2_O nanoparticles.

Chemical additives including sodium dodecyl sulfate (SDS), polyvinylpyrrolidone (PVP) and EDA have been often utilized in many solution-based synthesis as well as electrochemical synthesis of various nanostructures as a shape modifying agent [29,30,31]. However, the fabrication of Cu_2_O nanoparticles via electrodeposition using EDA as a size and shape modification additive has not been report to the best of our knowledge. Here, there are several factors that could influence the size, density and shape of the Cu_2_O nanoparticles in the presence of EDA. The chemical additive EDA has a tendency to adsorb on high energy faces of a growing crystal, thus leading to a controlled size and shape of the Cu_2_O nanoparticles. The drastic decrease in the nanoparticle size, with increase in the EDA content, can subsequently increase the density of the nanoparticles on the FTO substrate. The decrease in size and increase in the density was corroborated by the SEM data. However, the EDA present in the electrolytic bath can also interact with the FTO substrate occupying deposition sites of Cu^2+^ ions. This could hinder the deposition of Cu^2+^ and lower the deposition of the Cu_2_O on the FTO substrate. Such interaction of EDA with the FTO substrate resulted in lower sample thickness. This was confirmed by the XRD data and also verified by visual inspection. Thus, the presence of EDA affects the deposition forming small nanoparticle size, higher density and lower sample thickness with increase in EDA content. Further, the interplay between the applied potential, electrolyte pH and EDA content is still unclear and thus requires further exploration. Presently, detailed investigation is underway to understand the precise influence of EDA on size, density, deposition rate and conductivity of these Cu_2_O samples.

## 4. Conclusions

In conclusion, successful electrodeposition of Cu_2_O nanoparticle was performed in the presence of EDA. The nanoparticles size was varied between 54.09 nm to 966.97 nm, by adjusting the EDA content in the electrolyte bath. The absorption spectra indicated a blue shift in the absorption peak with decrease in the Cu_2_O nanoparticle size. The enzyme, GOx was successfully immobilized on the Cu_2_O nanoparticles. The sensitivity of the biosensor was influenced by the presence of EDA. The sensitivity increased with EDA content during electrodeposition. More detailed investigations elucidating the influence of EDA on the Cu_2_O nanoparticle size, density and sample thickness are underway.

## Figures and Tables

**Table 1:** Size distribution of Cu_2_O nanoparticles with varying EDA content.

Cu_2_O Sample with Varying EDA Content in mL	Particle Size (nm)	
0.2	966 ± 23	
0.4	792 ± 16	
0.8	802 ± 34	
1.0	54 ± 20	427 ± 20

**Table 2:** Comparison of glucose biosensor characteristics with different Cu_2_O nanostructures in literature.

Working Electrode	Sensitivity (μA/cm^2^·mM)	$K_{M}^{app}$ (mM)	LOD (μM)	Linear Range (mM)	Reference
GOx/Cu_2_O	55.32	0.79	0.2	0.2–3.5	The work
GOx/Cu_2_O/EDA (0.2)	1243.2	1.00	0.1	0.1–3.5	The work
GOx/Cu_2_O/EDA (1)	1538	1.25	0.1	0.1–3.5	The work
Cu_2_O/GNs	285	-------	3.3	0.3–3.3	[23]
Cu_x_O/Cu	1620	-------	49	0–4	[24]
Cu_2_O/CRG	-------	-------	1.2	0.1–1.1	[25]
Cu_2_O/Cu	62.29	-------	37	0.05–6.75	[26]
Cu_2_O/Carbon Vulcan XC-72	629	-------	2.4	0–6	[27]
Cu_2_O/Nafion/Glassy Carbon	121.7	-------	38	0–0.5	[28]
